# Comparison of Fascicular Turnover Flap and Autograft in a Rat Facial Nerve Model

**DOI:** 10.3390/jcm15082902

**Published:** 2026-04-10

**Authors:** Ivan Shpitser, Mark Gabriyanchik, Alexey Fayzullin, Yana Khristidis, Kamil Salikhov, Olesya Startseva, Olga Kolesnikova, Kirill Pirogov, Peter Timashev, Anna Vedyaeva

**Affiliations:** 1 Central Research Institute of Dentistry and Maxillofacial Surgery, Moscow 119021, Russia; 2 Department of Oncology, Radiotherapy and Reconstructive Surgery, Sechenov First Moscow State Medical University (Sechenov University), Moscow 119991, Russia; 3 Department for Business Development, Sechenov First Moscow State Medical University (Sechenov University), Moscow 119991, Russia; 4 Institute for Regenerative Medicine, Sechenov First Moscow State Medical University (Sechenov University), Moscow 119991, Russia; 5 N.V. Sklifosovsky Research Institute for Emergency Medicine of the Department of Health of Moscow, 3 Bolshaya Sukharevskaya Sq., Moscow 129090, Russia; 6 Institute of Clinical Medicine, Pirogov Russian National Research Medical University, 1/6 Ostrovitianova St., Moscow 117513, Russia

**Keywords:** facial nerve, rat model, fascicular turnover flap, autograft, whisker tracking, histomorphometry, peripheral nerve regeneration

## Abstract

**Background**: Fascicular turnover flap (FTF) is a donor-sparing option for segmental facial nerve repair. This study compared autologous nerve grafting with proximally based and distally based FTF in a rat facial nerve model. **Methods**: Adult male Wistar rats were randomized to autograft, proximal FTF, or distal FTF (n = 8 per group). A single additional animal with an untreated defect served as a qualitative histological reference. The prespecified primary endpoint was whisker motion amplitude at week 8; the secondary endpoints were central section histomorphometry (nerve tissue area, µm^2^) and variability metrics (IQR, SD, and coefficient of variation) as measures of reproducibility. Non-parametric tests (Kruskal–Wallis; Mann–Whitney U) were used; pairwise functional comparisons were Holm-corrected; and effect sizes were expressed as Cliff’s δ. **Results**: At week 8, the overall functional comparison was significant (Kruskal–Wallis *p* = 0.047), but no pairwise contrast remained significant after Holm correction. Functional recovery was highest in the autograft group, followed by proximal FTF and distal FTF. Both FTF groups showed lower inter-animal variability than autograft for the week-8 functional endpoint, with the distal FTF showing the lowest dispersion. Central section nerve area comparisons did not reach global significance; effect sizes and descriptive statistics favored autograft, and a single unadjusted pairwise contrast (autograft > proximal FTF) should be interpreted cautiously. **Conclusions**: Both FTF configurations achieved measurable functional and structural regeneration while avoiding an additional free donor nerve graft. Within an 8-week window, autograft remained the benchmark. Between FTF variants, distal FTF produced more stable functional outcomes, but this did not translate into superior functional recovery. Confirmation in larger, balanced cohorts with longer follow-up and vascular/neural labeling is warranted.

## 1. Introduction

Restoring the integrity of peripheral nerves remains one of the most demanding problems in reconstructive microsurgery. Traumatic injuries, tumor resections, and iatrogenic lesions of major peripheral and cranial nerves often lead to lasting functional and psycho-emotional impairment; when gaps preclude tension-free direct neurorrhaphy, outcomes are frequently suboptimal [1,2,3].

Autologous nerve grafting is the clinical gold standard for bridging extended defects because it provides native extracellular matrix and Schwann-cell scaffolding [1,2]. However, autografts are constrained by donor-site morbidity and scarcity, length limitations, and qualitative/quantitative mismatch when sensory donors are used to reconstruct motor or mixed nerves. This sensory–motor disparity has been shown to hinder motor regeneration in experimental settings [4].

To mitigate mismatch, “like-with-like” strategies have been explored. One concept is fascicular shifting (FS), in which a fascicular bundle is mobilized and redirected to bridge a defect while preserving modality and intraneural topography [5,6]. In preclinical work, FS performed at least as well as—and, for motor recovery metrics, sometimes better than—conventional sensory grafting; anatomical and early clinical observations further suggest feasibility in brachial plexus reconstruction [5,6,7].

Another approach is the fascicular turnover flap (FTF), where a portion of the parent nerve’s fascicles is rotated toward the defect without full transection of the main trunk. FTF offers a single coaptation site, maintains vascularity via intrafascicular microvessels, and avoids harvesting an additional donor nerve, principles rooted in supermicrosurgical practice [8,9,10]. Experimental studies indicate that, in rat models, FTF can yield functional and morphometric outcomes comparable to autograft in facial nerve reconstruction [11] and that distally based FTF may show more mature myelination and robust axonal regeneration than a classical autograft in a sciatic nerve gap [12]. Clinical feasibility has also been demonstrated for reconstruction of a sural nerve defect after biopsy [10], though reports caution about neuroma formation—including neuroma-in-continuity—at the fascicular separation/coaptation site; this should inform patient selection and revision strategies [13].

For facial nerve reconstruction, the requirements are stricter: accurate topography and synchronous motor reinnervation are essential to minimize asymmetry and synkinesis. Against this background, FTF is theoretically attractive for preserving intraneural orientation and vascularity while minimizing donor morbidity. Nevertheless, comparative data specific to the facial nerve remain limited, and results vary across models and flap orientations [8,11,14,15].

### 1.1. Study Focus and Rationale: Effectiveness and Reproducibility

Beyond central tendency alone, clinical translation demands reproducible techniques that deliver consistent outcomes across subjects. Because FTF is performed on the parent nerve and aims to preserve local vascularity, it was hypothesized that its inter-animal variability (e.g., IQR, SD, and coefficient of variation) might be lower—i.e., more reliable/standardizable—than that of conventional grafting and that flap orientation (proximal vs distal) could further influence recovery magnitude and dispersion.

Supporting this expectation, Choi et al. [12] reported that a distally based FTF produced more mature myelination and robust axonal regeneration than autograft in a rat sciatic nerve model, attributing the advantage to preserved intrinsic vascularization of the distal pedicle. Additionally, the distal configuration maintains the native orientation of endoneurial tubes in the segment closest to the target musculature, which may facilitate axonal entry into existing Schwann-cell columns.

### 1.2. Goal

Our goal was to compare functional and morphologic outcomes and their variability for three reconstructions of a segmental facial nerve defect in rats: autograft (reference benchmark), proximal FTF, and distal FTF. The primary aim was to compare the two FTF configurations with respect to recovery and reproducibility, with autograft serving as a clinical standard for context. It was hypothesized that FTF would achieve recovery comparable to autograft, and that the distal FTF might demonstrate a trend toward superior outcomes with comparable or lower variability. Whisker motion amplitude at week 8 was the prespecified primary endpoint; histomorphometry (nerve tissue area and cell density) constituted secondary endpoints, and group variability metrics (IQR/median; SD/mean) were analyzed as measures of reproducibility.

## 2. Materials and Methods

### 2.1. Experimental Model

Adult male Wistar rats (250–300 g) from the vivarium of the Sechenov First Moscow State Medical University were housed at 22 ± 1 °C on a 12 h light/dark cycle with ad libitum access to food and water. All procedures complied with ETS-123 and were approved by the Institutional Ethics Committee. The animals were randomly allocated (sealed envelope method) to three groups (autograft, proximal FTF, and distal FTF; n = 8 per group). The sample size was based on previous FTF studies using comparable functional endpoints in rat models [11,12]. One additional animal with an untreated defect served as a qualitative histological reference (Group IV) and was not included in comparative analyses. The operating surgeon was not blinded to group assignment owing to the nature of the interventions. Statistical analysis was performed without knowledge of group labels (coded datasets). To assess reproducibility, inter-animal variability (SD, IQR, and coefficient of variation) was recorded for each metric and compared across groups.

Anesthesia was induced intraperitoneally with tiletamine–zolazepam (Zoletil 40; 40 mg/kg) and xylazine (5 mg/kg). Adequacy was verified by loss of the paw pinch reflex. Body temperature was maintained at ~37 °C using a thermostatic heating pad (RWD Life Science, Shenzhen, China). Analgesia: An amount of 1.0 mg/kg s.c. of meloxicam was administered once daily for 3 days. A single perioperative prophylactic dose of enrofloxacin of 10 mg/kg s.c. was administered. The surgical field was shaved and prepared with povidone–iodine. A sterile drape was applied.

### 2.2. Operative Access and Modeling of Nerve Defect

**Figure 1:**
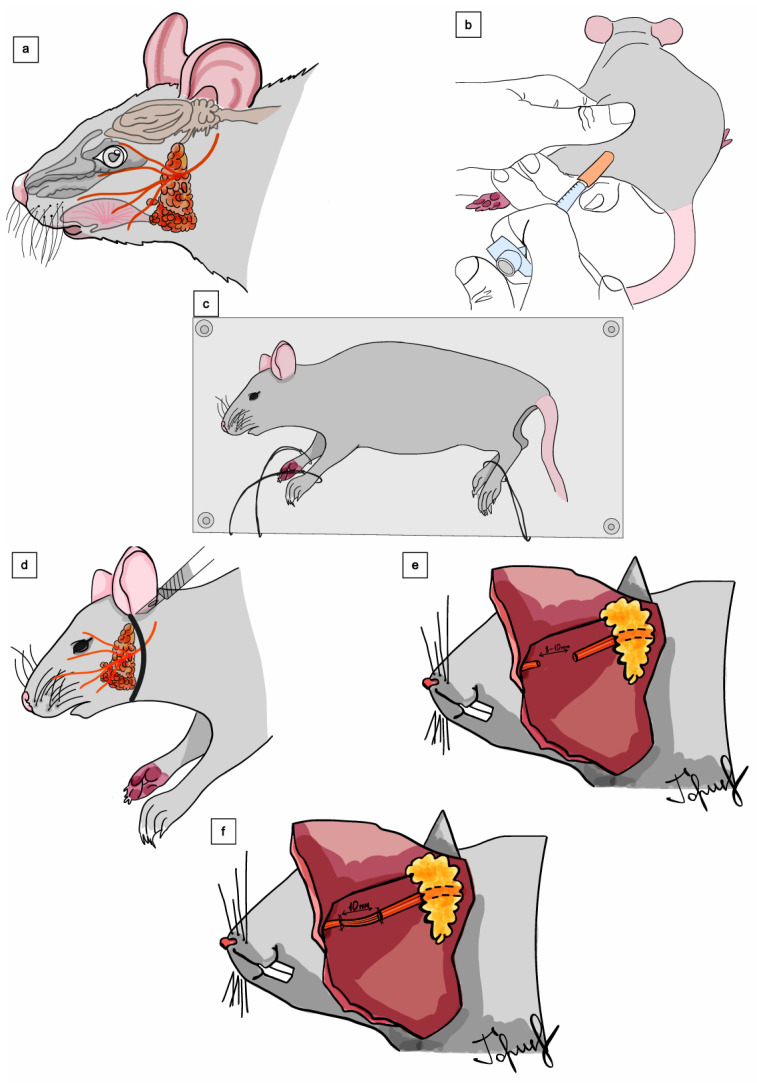

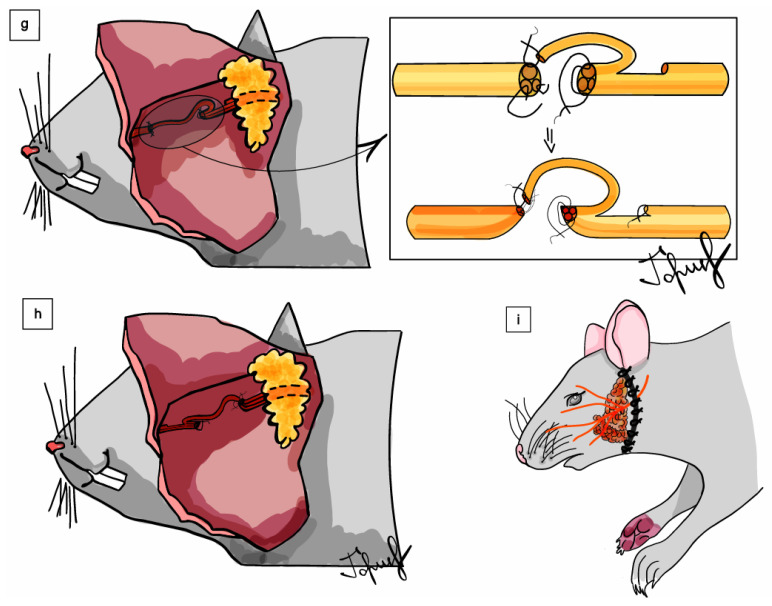
Schematic illustration of the experimental design and surgical procedure of facial nerve reconstruction using a fascicular turnover flap in a rat model. (**a**) Surface anatomy of the rat facial region with schematic projection of the facial nerve branches and parotid gland. (**b**) Perioperative handling and anesthetic administration. (**c**) Positioning and fixation of the animal on the operating platform before microsurgical exposure. (**d**) Surgical exposure of the facial nerve branches in the parotid region. (**e**) Creation of a 10 mm defect in the marginal mandibular branch after resection of the target segment. (**f**) Preparation of the fascicular turnover flap from the proximal nerve stump. (**g**) Rotation of the fascicular flap into the defect and microsurgical neurorrhaphy; inset demonstrates the principle of epineurial suturing. (**h**) Final positioning of the fascicular turnover flap after reconstruction of nerve continuity. (**i**) Wound closure following completion of the reconstructive stage. The schematic illustration of the procedure reflects the fascicular turnover flap technique as originally described by Uehara et al. [11].

A preauricular–mandibular skin incision was made along the line between the external auditory meatus and the mandibular angle. The parotid gland was gently retracted laterally to expose the main facial nerve trunk and its buccal and marginal mandibular branches. Under an operating microscope (Leica M525 F20, up to ×25), the marginal mandibular branch was isolated, and a 10 mm segmental gap was created with microshears between clearly defined proximal and distal stumps. The course of manipulations is shown in Figure 1.

### 2.3. Reconstruction Techniques

All reconstructions were performed under high magnification using microsurgical instruments (Aesculap, Tuttlingen, Germany). Tissues were irrigated with sterile saline to avoid desiccation; the closure was layered (6-0 Vicryl for glandular/soft tissues; 4-0 nylon for skin).

Group I—autograft (control): The defect of the marginal mandibular branch was reconstructed using an autologous nerve graft harvested from the buccal branch and coapted end-to-end with interrupted 10-0 epineurial nylon sutures.

Group II—proximal fascicular turnover flap (proximal FTF): A ~10-mm fascicular segment on a pedicle was mobilized from the proximal stump, rotated distally, and coapted to the distal stump, preserving continuity (single coaptation site) and putative intrafascicular perfusion. Fixation: 1–2 interrupted 10-0 epineurial sutures.

Group III—distal fascicular turnover flap (distal FTF): A comparable fascicular segment was mobilized from the distal stump, rotated proximally, and coapted to the proximal stump (single coaptation).

All reconstructions were performed by the same microsurgeon to minimize operator-related variability and ensure procedural reproducibility.

### 2.4. Postoperative Follow-Up

The animals were housed individually with environmental enrichment and monitored daily for 8 weeks for wound status, self-mutilation, infection, and weight. All 25 animals completed follow-up (no unplanned deaths).

The predefined humane endpoints included >20% body weight loss, persistent self-mutilation, wound dehiscence unresponsive to treatment, or signs of systemic infection. No animal reached a humane endpoint.

### 2.5. Functional Assessment (Whisker Movements)

Whisker movement amplitude is a validated functional endpoint for facial nerve regeneration in rats [11,16]. Whisker movements were recorded every week in awake animals after ≥5 min of habituation, using a high-speed video at 240 fps for 15 s epochs with the camera perpendicular to the muzzle. Recordings were acquired at a consistent time of day under standardized lighting. The most anterior vibrissa on the operated side (and the contralateral homologue for reference) served as fiducials. Angles were measured in ImageJ 1.54 relative to a fixed facial landmark axis. Two animals (one each from Groups II and III) were excluded from the functional analysis based on prespecified video quality criteria (obscured vibrissae), yielding n = 8/7/7. For each epoch, the following was obtained: min. (rest angle), max. (maximal protraction), and Δ = max − min (amplitude). Analyses were performed on the operated side, and Δ was also expressed as a percentage of the contralateral side. Video scoring was blinded to group and timepoint. For each group, both the central tendency (median amplitude) and dispersion (IQR, SD, and coefficient of variation) were calculated as indicators of functional reproducibility.

### 2.6. Histomorphological Assessment

**Figure 2:**
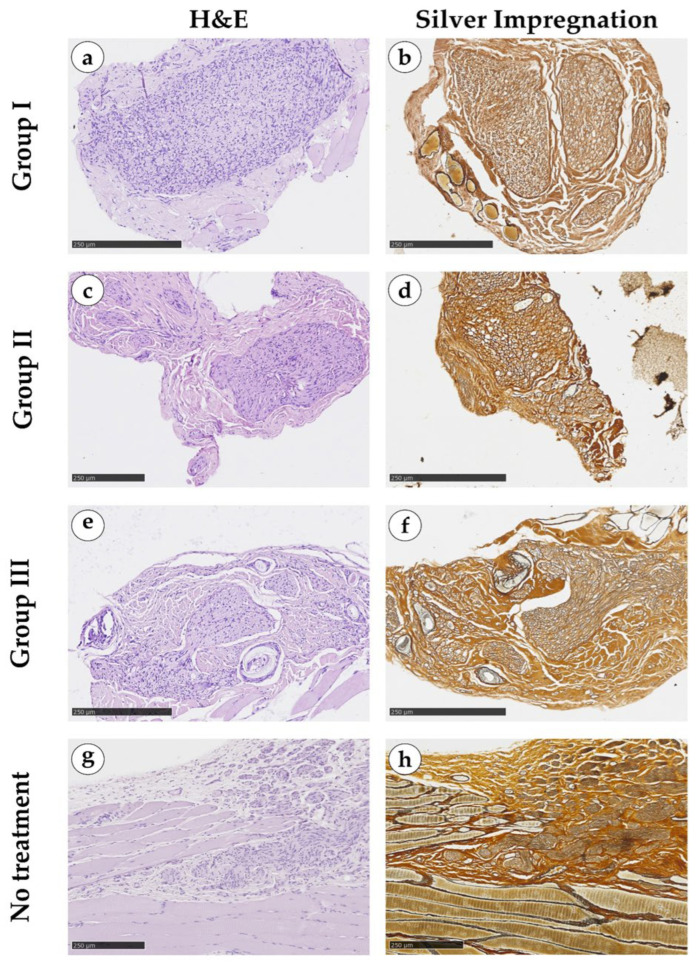
Micrographs of the central sections. (**a**,**b**) Group I (autograft); (**c**,**d**) Group II (proximal FTF); (**e**,**f**) Group III (distal FTF); and (**g**,**h**) Group IV (without treatment). (**a**,**c**,**e**,**g**) H&E; (**b**,**d**,**f**,**h**) silver impregnation.

Sections with a thickness of 4 μm of the formalin-fixed, paraffin-embedded tissue samples were stained with hematoxylin and eosin (H&E) and by silver impregnation for the detection of nerves. The slides were digitized in the ×400 magnification mode using a NanoZoomer S20MD scanner (Hamamatsu, Saitama, Japan) for morphological analysis. The area of nerve tissue was evaluated in the central sections of the defects and was measured using QuPath v.0.4.3 (Figure 2).

### 2.7. Statistical Analysis

The main morphometric analysis used central sections from Groups I–III. Distributional assumptions were assessed with the Shapiro–Wilk test. Non-normal data were analyzed using the Kruskal–Wallis test followed by prespecified Mann–Whitney U contrasts (I–II, I–III, and II–III). Effect sizes were expressed as Cliff’s δ with 95% bootstrap confidence intervals (10,000 resamples; fixed seed). The results are reported as medians [IQR], with means ± SD provided descriptively. Intra-group variability (IQR, SD, and coefficient of variation) was calculated as a measure of reproducibility. The primary morphological indicator was the total area of nervous tissue (µm^2^); fiber density (number/mm^2^) was also computed. Given unequal counts of evaluable central sections (I = 13, II = 5, and III = 8), a balanced 5–5–5 subsampling robustness check (2000 iterations; fixed seed) was performed to verify consistency. Unequal counts reflected variability in the number of technically evaluable central sections obtained after tissue processing and sectioning. Histomorphometric comparisons were performed at the section level; therefore, section-based analyses were interpreted as exploratory and were complemented by a balanced subsampling robustness check. Functional recovery (Δ whisker amplitude) at week 8 was the prespecified primary endpoint; the same statistical pipeline was applied, with Holm correction for multiple pairwise comparisons. Longitudinal trends (weeks 1–8) were examined descriptively. Analyses were conducted in Python 3.12 (SciPy and pandas) and SPSS 26; figures were created with Matplotlib 3.9.0 and GraphPad Prism 9.

### 2.8. Ethical Aspects

All procedures complied with institutional and international guidelines for the care and use of laboratory animals and were approved by the Local Ethics Committee of I.M. Sechenov First Moscow State Medical University (Sechenov University) (protocol code: 10-24; approval date on 14 April 2024). The study design followed the principles of the 3Rs (replacement, reduction, and refinement) and included reproducibility analysis to maximize information gain from a minimal number of animals (Supplementary Materials).

## 3. Results

### 3.1. Functional Assessment (Whisker/Vibrissae Movements)

The main criterion for recovery was the amplitude of the vibrissae movements (Δ (max–min); degrees). The prespecified primary functional endpoint was whisker movement amplitude at week 8 after surgery; the data from weeks 1–7 are presented descriptively (see the Section 4).

The prespecified week-8 functional comparison showed a significant overall difference among the three groups (Kruskal–Wallis H = 6.11; *p* = 0.047). However, in the pairwise Mann–Whitney U tests, no comparison remained significant after Holm correction (all q > 0.05). A nominal difference was observed between the autograft and distal FTF groups (U = 48; *p* = 0.021), but this did not retain significance after correction (q = 0.062).

At week 8, the mean (±SD) whisker movement amplitudes were 21.12 ± 13.18° for the autograft group, 14.94 ± 8.52° for the proximal FTF group, and 9.35 ± 4.23° for the distal FTF group. The corresponding median [Q1–Q3] values were 19.21 [11.82–26.33]°, 14.78 [9.38–17.22]°, and 8.12 [7.05–10.39]°. The dispersion analysis showed IQRs of 14.51°, 7.84°, and 3.33°, and coefficients of variation of 0.62, 0.57, and 0.45 for the autograft, proximal FTF, and distal FTF groups, respectively (n = 8/7/7).

**Figure 3:**
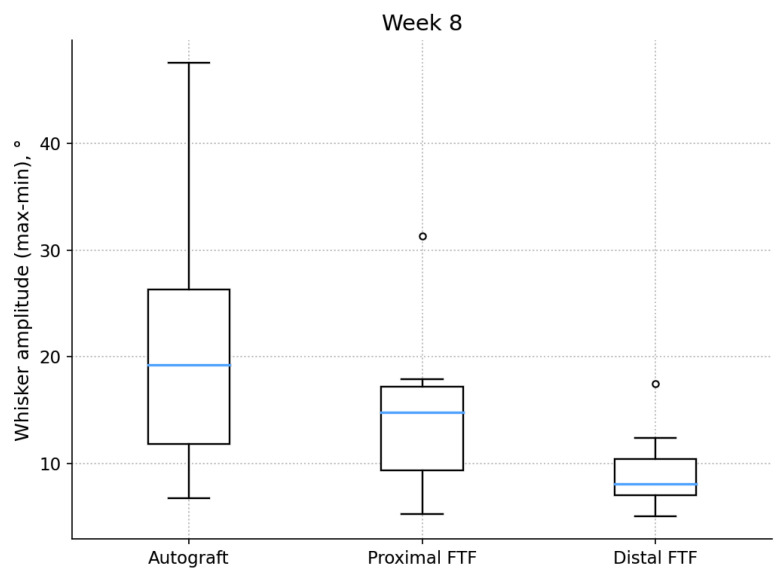
The amplitude of the movements of the vibrissae in the 8th week by groups. Each box shows the interquartile range, horizontal line—median, and whiskers—1.5 × IQR. n = 8/7/7.

Throughout the observation period, the whisker movement amplitude increased in all groups. No statistically significant between-group differences were detected at weeks 1–7 (all *p* > 0.05). By week 8, the ordering of group means was autograft > proximal FTF > distal FTF (Figure 3; Table 1).

### 3.2. Morphology (Qualitative Assessment)

Group I (autografts) demonstrated the most favorable structural characteristics. Most specimens contained peripheral nerves with typical histological architecture and staining patterns. Intense Schwann cell proliferation and migration were evident, predominantly within the larger autografted nerves, forming clusters of multidirectional spindle-shaped cells. No marked signs of nerve degeneration were observed. Quantitative assessment confirmed that this group exhibited the highest mean values for total nerve tissue area per section and for cell density within the neural structures.

Group II (proximal FTF) displayed a generally preserved nerve architecture, with more frequent signs of reparative regeneration than in the autografts group, such as individual nerve processes extending beyond the main nerve. A tendency toward reduced total nerve area compared with Group I was noted, suggesting a lower regenerative potential associated with this treatment.

Group III (distal FTF) showed morphological features largely comparable to those of the proximal FTF group. However, the Schwann cell proliferation was less pronounced. Several samples contained numerous medium-sized nerves in addition to large trunks, possibly reflecting structural heterogeneity and variations in functional reinnervation within the distal portion of the flap.

Group IV (untreated-defect reference group) was assessed qualitatively only and was not included in the randomized comparative analyses, predominantly consisting of connective tissue interspersed with residual muscle fragments. Foci of post-injury repair and mild immune cell infiltration were occasionally present in muscle regions. Limited peripheral nerve regeneration was observed only in a single sample, represented by a small cluster of multidirectional Schwann cells at the edge of the defect area.

### 3.3. Quantitative Assessment (Central Sections)

After 8 weeks, the total area of nerve tissue (µm^2^) was measured on evaluable central sections (not animals).

Because the data were not normally distributed and group sizes were unequal (Group I = 13, Group II = 5, and Group III = 8), non-parametric tests were applied. The overall comparison among the three groups did not show a significant difference (Kruskal–Wallis H = 5.51; *p* = 0.063).

In prespecified pairwise Mann–Whitney tests, the autograft group showed a larger nerve tissue area than the proximal FTF group (U = 55, *p* = 0.026, and Cliff’s δ = +0.69); however, this finding should be interpreted cautiously in the context of the non-significant overall Kruskal–Wallis test and unequal section counts. No significant difference was observed between the autograft and distal flap groups (U = 67, *p* = 0.301, and δ = +0.29) or between the two fascicular turnover flap groups (U = 10, *p* = 0.171, and δ = −0.50).

**Figure 4:**
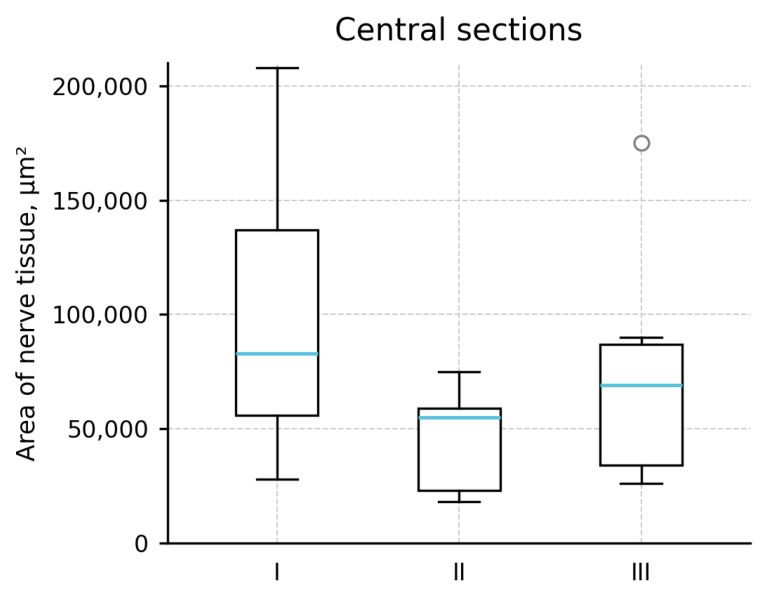
Area of nerve tissue in central sections. Box = IQR; line = median; whiskers = 1.5 × IQR. Group labels I–III correspond to autograft, proximal FTF, and distal FTF.

Descriptive statistics (median [IQR], µm^2^): Group I (Autograft): 82,623 [55,644–137,906]; Group II (Proximal FTF): 54,347 [22,166–58,198]; and Group III (Distal FTF): 70,229 [32,862–86,233]. The coefficients of variation (SD/mean) were 0.57, 0.55, and 0.67 for Groups I–III, respectively. The section–animal variabilities expressed as IQRs were 82,262 µm^2^, 36,032 µm^2^, and 54,598 µm^2^ for Groups I–III. No outliers were excluded from the analysis (Figure 4; Table 2).

## 4. Discussion

Throughout the 8-week observation period, the whisker movement amplitude increased progressively in all groups. No statistically significant differences were found at weeks 1–7 (*p* > 0.05). By week 8, the autograft group showed the highest recovery amplitude, whereas both fascicular turnover flap groups demonstrated measurable, though lower, recovery within the same time frame. Gross examination at 8 weeks revealed no neuroma-like tissue or excessive scarring at the coaptation site in any group.

At week 8, the overall between-group comparison reached statistical significance, but no pairwise contrast remained significant after Holm correction. Central section histomorphometry did not differ significantly, although effect sizes favored autograft. For the week-8 functional endpoint, both FTF groups had narrower IQRs and lower coefficients of variation than the autograft group, indicating more consistent recovery across animals within each group.

Because the histomorphometric analysis was based on unequal numbers of evaluable central sections (13, 5, and 8), balanced subsampling was performed to assess robustness. Subsampling yielded results consistent with the primary analysis: global differences were infrequent, and pairwise contrasts rarely reached *p* < 0.05; the median Cliff’s δ values preserved the same overall direction (with autograft tending to exceed FTF in section-based morphometric outcomes). These findings support that the observed pattern was not driven by group imbalance.

The present results align with, yet also temper, previously reported outcomes. In a rat facial nerve model, Uehara et al. described comparable functional and morphometric regeneration between FTF and autograft, supporting the applicability of a vascularized fascicular segment for motor branches [11]. In a sciatic nerve defect model, Choi et al. reported a morphologic advantage of distally based FTF over autograft, attributed to preserved vascularization and reduced ischemia within the flap [12]. Conceptually related fascicular shifting (FS) studies have demonstrated equivalent or improved results compared with traditional grafting while minimizing donor morbidity [9,10].

**Figure 5:**
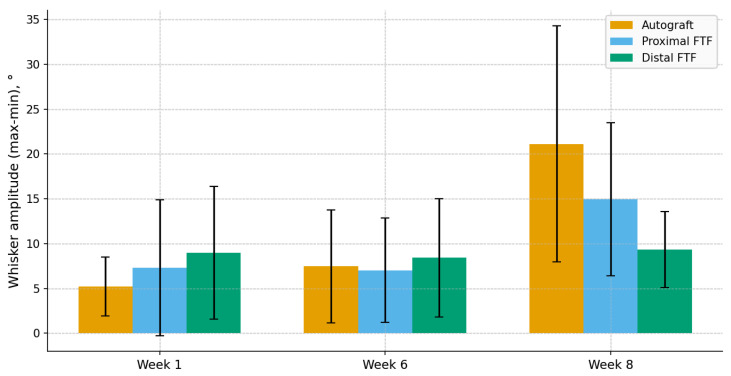
The dynamics of the amplitude of the vibrissae (mean ± SD) at the 1st, 6th and 8th weeks.

Against this background, the present 8-week endpoint provides a more conservative picture: functionally, autograft achieved the highest amplitude, whereas both FTF configurations achieved measurable but lower recovery; histomorphometry did not differ significantly. Several factors may explain these findings. Some previous rodent studies assessed longer endpoints (~12 weeks) [11], when myelin maturation and vascular remodeling can more clearly reflect the benefits of vascularized constructs [17,18,19]. Consistent with this, early timepoints in our series showed similar amplitudes across groups, with divergence emerging by week 8 (Figure 5). A longer follow-up might, therefore, attenuate or reverse the observed difference.

Anatomical and mechanical factors may also influence outcomes. The marginal mandibular branch of the facial nerve differs from the sciatic nerve in diameter, fascicular composition, and surrounding tissue tension. A distally based FTF may experience bending or stretching of the vascular pedicle after rotation; a 180° turnover can impose curvature or mild tension, particularly in the distal configuration, potentially compromising inflow or outflow.

These factors could account for the lower Δ observed in the distal flap, although vascular parameters were not directly assessed in this study.

Notably, the distal FTF showed a higher median nerve tissue area than the proximal FTF, yet lower functional recovery. This structure–function dissociation suggests that tissue bulk alone is not a reliable surrogate for motor outcomes: a 180° fascicular rotation may compromise axonal directionality and topographic alignment, resulting in more tissue but less effective reinnervation of target musculature.

The comparative analysis of dispersion metrics indicates that both FTF variants provided stable and reproducible outcomes across animals, with the distal configuration showing the lowest variability.

Methodological limitations should also be considered. The modest sample size and unequal numbers of analyzable samples reduce power for morphometric endpoints. Sections were evaluated with basic stains; immunohistochemical and vascular labeling (e.g., S-100, NF-200, CD31) were not performed, limiting mechanistic inference on Schwann cell phenotype and microvascularity. Electrophysiological assessment (e.g., compound muscle action potential) was not included, precluding direct comparison with studies that used CMAP as a primary outcome [11].

Although prior studies suggested that vascularized FTF configurations may match or even exceed autograft under selected experimental conditions, the present data indicate that this advantage is not uniform across nerve models, flap orientations, and follow-up windows. In the facial nerve, where topographic precision and coordinated motor reinnervation are especially important, preservation of vascularity alone may be insufficient to ensure superior early functional recovery. Thus, the present study refines the interpretation of earlier work: FTF should be viewed not as a universal replacement for autograft, but as a donor-sparing reconstructive option whose main potential advantages may lie in procedural reproducibility, avoidance of donor site morbidity, and applicability in selected short-gap settings.

From a reconstructive perspective, FTF may be considered in several clinical scenarios. First, when expendable sensory donors (e.g., sural nerve) are unavailable or their harvest would cause unacceptable morbidity, FTF eliminates the need for a separate donor site. Second, for short-to-moderate gap lengths amenable to fascicular rotation without excessive tension, FTF avoids the size and modality mismatch inherent in interposition grafting. Third, in compromised or previously irradiated wound beds, the preserved continuity with the parent trunk and its intrinsic microvascular supply may offer a biological advantage over a free, avascular autograft [17,18,19]. These potential indications notwithstanding, FTF sacrifices a portion of the donor nerve trunk, and the 180° fascicular rotation may compromise axonal directionality; applicability to longer gaps or polyfascicular nerves remains unproven. However, clinicians should consider the possibility of neuroma formation at the fascicular separation or coaptation site [13].

Careful selection of indications, tension-free coaptation, and avoidance of blind fascicular endings are recommended.

## 5. Conclusions

The overall week-8 functional comparison reached statistical significance, but pairwise differences did not remain significant after Holm correction. The proximal FTF occupied an intermediate position, while the distal FTF showed more stable outcomes; however, this did not translate into superior functional recovery within the 8-week observation window. According to the morphometric analysis of central sections (nerve tissue area and fiber density), a nominal trend favoring autograft was observed, but the differences were not statistically confirmed. No statistically significant differences were identified between proximal and distal FTF in pairwise comparisons. Within each reconstruction technique, no significant differences were detected among section locations (central/proximal/distal).

Overall, fascicular turnover flaps provided measurable regeneration while avoiding an additional free donor nerve graft and showed acceptable reproducibility. Between the two FTF configurations, the distal flap demonstrated more stable functional outcomes, whereas the autograft remained the benchmark within this 8-week observation window.

Future studies should increase sample size and extend follow-up to ≥12 weeks. Standardized assessment of flap vascularization (e.g., CD31 immunolabeling or microangiography) and neural markers (S-100, NF-200), as well as control of technical parameters (rotation length, angle, and pedicle tension), may help clarify the mechanisms underlying fascicular flap performance and its long-term potential.

These findings suggest that reproducibility may be a potential advantage of fascicular turnover flaps, particularly for the week-8 functional endpoint, and warrant further study.

## Figures and Tables

**Table 1:** Pairwise comparisons of function at week 8 (Δ, degrees).

Comparison	U	*p*-Value	Cliff’s δ	95% CI	Effect
I vs. II	37	0.336	+0.32	[−0.29, +0.86]	Small
I vs. III	48	0.021	+0.71	[+0.25, +1.00]	Large
II vs. III	37	0.128	+0.51	[−0.10, +1.00]	Large

Note: Unadjusted pairwise *p*-values are reported. No comparison remained statistically significant after Holm correction for multiple comparisons.

**Table 2:** Pairwise comparisons of histomorphometry (central sections; groups I–III).

Comparison	U	*p*-Value	Cliff’s δ	95% CI	Effect
I vs II	55	0.026	+0.690	[+0.26, +1.00]	large
I vs III	67	0.301	+0.288	[−0.25, +0.75]	small
II vs III	10	0.171	−0.500	[−0.95, +0.10]	large

Note: Unadjusted pairwise *p*-values are reported. No comparison remained statistically significant after Holm correction for multiple comparisons.

## Data Availability

The raw data supporting the conclusions of this article will be made available by the authors upon request.

## Supplementary Materials

The following supporting information can be downloaded at https://www.mdpi.com/article/10.3390/jcm15082902/s1: Table S1: ARRIVE checklist.

## Abbreviations

The following abbreviations were used in this manuscript:

Δ	Whisker amplitude (max–min), degrees
CD31	Cluster of differentiation 31
Cliff’s δ	Cliff’s delta (effect size)
ETS-123	European Convention for the Protection of Vertebrate Animals used for Experimental and other Scientific Purposes
FTF	Fascicular turnover flap
FS	Fascicular shifting
H&E	Hematoxylin and eosin
IQR	Interquartile range
i.p.	Intraperitoneal
KW	Kruskal–Wallis test
MWU	Mann–Whitney U test
NF-200	Neurofilament-200 (neuronal marker)
P/C/D	Proximal/central/distal (histology slice location)
S-100	S-100 protein (Schwann cell marker)
SD	Standard deviation
SPSS	Statistical Package for the Social Sciences
